# Comparative transcriptome analysis revealing the potential mechanism of seed germination stimulated by exogenous gibberellin in *Fraxinus hupehensis*

**DOI:** 10.1186/s12870-019-1801-3

**Published:** 2019-05-15

**Authors:** Qiling Song, Shuiyuan Cheng, Zexiong Chen, Gongping Nie, Feng Xu, Jian Zhang, Mingqin Zhou, Weiwei Zhang, Yongling Liao, Jiabao Ye

**Affiliations:** 1 College of Horticulture and Gardening, Yangtze University, Jingzhou, 434025 Hubei China; 2 National R&D for Se-rich Agricultural Products Processing Technology, Wuhan Polytechnic University, Wuhan, 430023 China; 3 Research Institute for Special Plants, Chongqing University of Arts and Sciences, Chongqing, 402160 China; 4 Engineering Research Center of Ecology and Agricultural Use of Wetland (Ministry of Education), Yangtze University, Jingzhou, 434025 Hubei China

**Keywords:** *Fraxinus hupehensis*, Seed germination, Transcriptome, Germination, Exogenous gibberellin, Differentially expressed genes

## Abstract

**Background:**

*Fraxinus hupehensis* is an endangered tree species that is endemic to in China; the species has very high commercial value because of its intricate shape and potential to improve and protect the environment. Its seeds show very low germination rates in natural conditions. Preliminary experiments indicated that gibberellin (GA_3_) effectively stimulated the seed germination of *F. hupehensis*. However, little is known about the physiological and molecular mechanisms underlying the effect of GA_3_ on *F. hupehensis* seed germination.

**Results:**

We compared dormant seeds (CK group) and germinated seeds after treatment with water (W group) and GA_3_ (G group) in terms of seed vigor and several other physiological indicators related to germination, hormone content, and transcriptomics. Results showed that GA_3_ treatment increases seed vigor, energy requirements, and trans-Zetain (ZT) and GA_3_ contents but decreases sugar and abscisic acid (ABA) contents. A total of 116,932 unigenes were obtained from *F. hupehensis* transcriptome. RNA-seq analysis identified 31,856, 33,188 and 2056 differentially expressed genes (DEGs) between the W and CK groups, the G and CK groups, and the G and W groups, respectively. Up-regulation of eight selected DEGs of the glycolytic pathway accelerated the oxidative decomposition of sugar to release energy for germination. Up-regulated genes involved in ZT (two genes) and GA_3_ (one gene) biosynthesis, ABA degradation pathway (one gene), and ABA signal transduction (two genes) may contribute to seed germination. Two down-regulated genes associated with GA_3_ signal transduction were also observed in the G group. GA_3_-regulated genes may alter hormone levels to facilitate germination. Candidate transcription factors played important roles in GA_3_-promoted *F. hupehensis* seed germination, and Quantitative Real-time PCR (qRT-PCR) analysis verified the expression patterns of these genes.

**Conclusion:**

Exogenous GA_3_ increased the germination rate, vigor, and water absorption rate of *F. hupehensis* seeds*.* Our results provide novel insights into the transcriptional regulation mechanism of effect of exogenous GA_3_ on *F. hupehensis* seed germination. The transcriptome data generated in this study may be used for further molecular research on this unique species.

**Electronic supplementary material:**

The online version of this article (10.1186/s12870-019-1801-3) contains supplementary material, which is available to authorized users.

## Background

*Fraxinus hupehensis* Chu, Shang et Su*.* is a woody plant of the Oleaceae family that is officially listed as a national rare and endangered tree species in China [1–3]. The species has high commercial value due to its slow growth, interlaced roots, intricate tree shape, and easy to shape. Furthermore, *F. hupehensis* has great potential to improve and protect the environment on account of its strong adaptability to arid and cold climates and resistance to diseases and insects [4, 5]. Previous studies have focused on the resource investigation [2], seedling technology [6], tissue culture [7], chemical composition, and pharmacological value of *F. hupehensis* [8]. In general, the fruit of *F. hupehensis* produces seeds after ripening and falling off in the spring of the second year. Under conventional sowing conditions, *F. hupehensis* seeds take more than 1 year of dormancy to germinate [9]. Considering the rapid development of the bonsai industry of *F. hupehensis* in China, resulting in serious damage to wild resources and a sharp decline in population. Therefore, its resources should be protected, and its reproductive and growth cycles should be accelerated through artificial technologies.

Seed germination refers to the resumption of embryo growth after water absorption and seed expansion until the radicle breaks through the endosperm and seed coat before germination. When stimulated by germination conditions (e.g., light, low temperature, water, and hormones), immature seeds ripen to form mature seeds and undergo germination [10]. Woody-plant seeds are naturally difficult to germinate, and dormancy is disrupted by external stimulation to promote germination. Exogenous hormones could promote the germination of dormant seeds. In particular, gibberellin **(**GA_3_) could break seed dormancy and play an endogenous signaling role during seed germination [11, 12]. In recent years, great advances have been achieved in the mechanism of GA_3_ on seed germination. GA_3_ plays two important roles in plant seed germination: (1) it overcomes the mechanical constraints imposed by seed mulch by weakening the tissues around the radicle [13], and (2) it increases the growth potential of embryos [14]. GA_3_ induces the degradation of the plant growth inhibitor DELLA protein by binding to its receptor, thereby promoting plant germination [15, 16]. The amounts of some hormones in the seeds affect the gene expression required for germination [13], and most of the genes involved in GA_3_ biosynthesis are up-regulated during seed germination [17]. Transcriptome methods have been used to study the gene expression of plant seeds during dormancy [18–20]. Transcriptome analysis reveals that the genes of many GA_3_ response elements are differentially expressed in *Arabidopsis* seeds with different dormancy levels [21]. In our recent work has shown that the seed germination of *F. hupehensis* was substantially promoted by GA_3_ treatment, followed by cryogenic stratification [22]. However, the physiological and molecular mechanisms by which GA_3_ promotes *F. hupehensis* seed germination remain unclear.

In this study, we constructed three independent cDNA libraries of three treated *F. hupehensis* seeds, including a CK group (dormant seeds, CK1, CK2, and CK3), a W group (germinated seeds after treatment with water, W1, W2 and W3), and a G group (germinated seeds after treatment with GA_3_, G1, G2, and G3), for Illumina HiSeq sequencing. We compared the seed vigor of these groups, as well as some physiological indicators related to their germination, hormone content, and transcriptomics among these seed groups. We also identified differentially expressed genes (DEGs) and transcription factors (TFs) related to seed germination and validated the expression genes involved in seed germination by qRT-PCR. Our data revealed that the physiological and transcriptomic aspects of the promotive effect of GA_3_ on *F. hupehensis* seed germination and provide insights into the involvement of GA_3_ in the seed germination mechanism of woody plants.

## Results

### Effects of GA_3_ treatment on physiological indexes during *F. hupehensis* seed germination

**Fig. 1:**
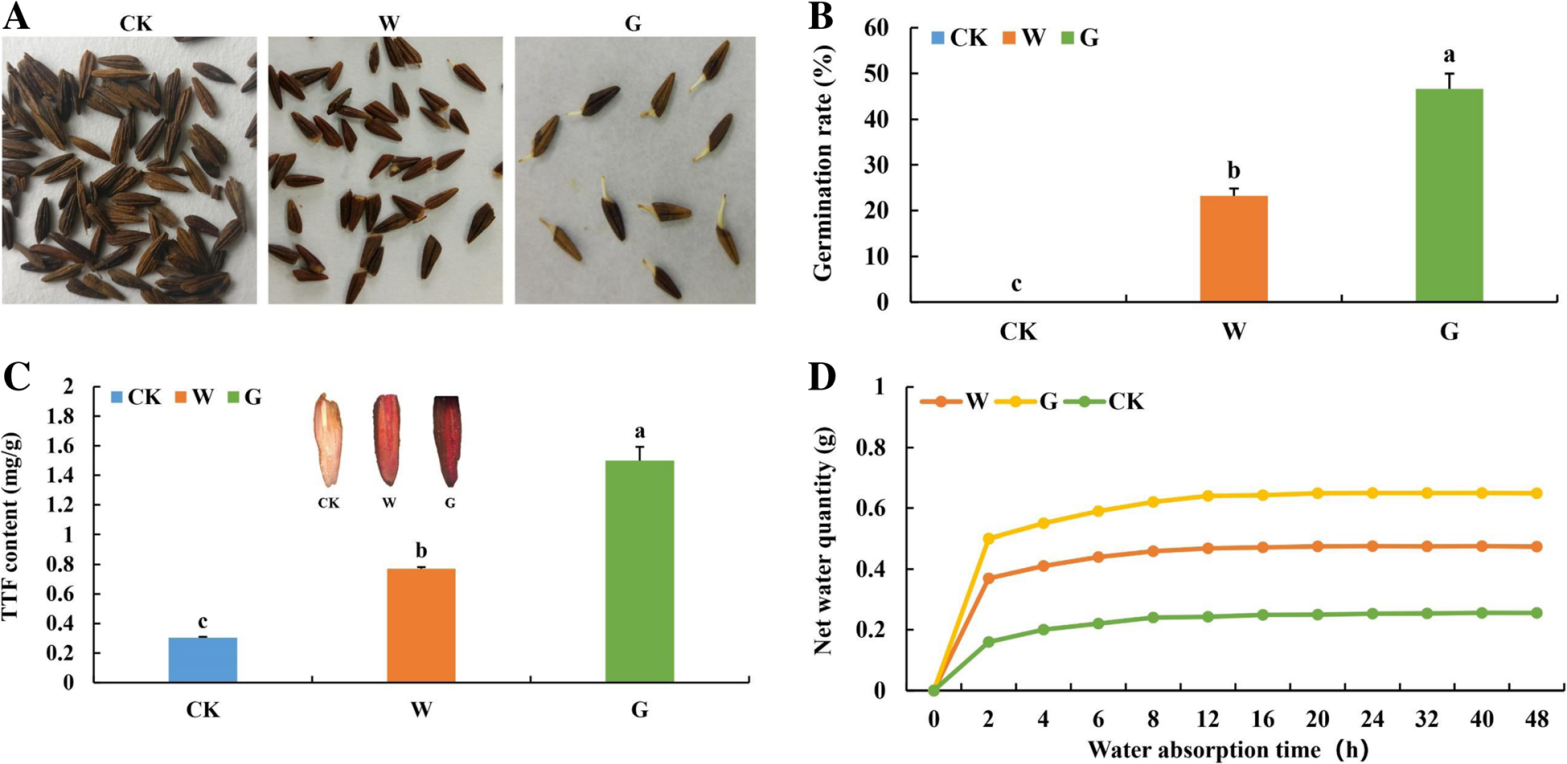
Comparison of physiological indexes during the germination of *F. hupehensis* seeds treated by three methods. CK, W and G represent dormant seeds, germinated seeds treated with water, and germinated seeds treated with GA_3_, respectively. **a** Comparison of *F. hupehensis* seed germination under three treatments. **b** Comparison of *F. hupehensis* seed germination rates under three treatments. **c** Comparison of *F. hupehensis* seed vigor under three treatments. **d** Comparison of *F. hupehensis* seed water absorption rates under three treatments. Data were analyzed by SPSS, followed by Duncan’s honestly significant difference test at *p* ≤ 0.05. All Statistical analyses of data had three biological repeats

In this study, the germinated seeds were obtained by low-temperature stratification after treatment with water (W group) or GA_3_ (G group); whereas the CK group comprised dormant seeds. After 50 days of treatment, the germination rates of water- and GA_3_-treated seeds were 46.67 and 26.03%, respectively. The CK seeds showed no signs of germination (Fig. 1a and b). Thus, GA_3_ and low temperature treatment significantly increased the seed germination rate of *F. hupehensis*. Triphenyltformazan (TTF) staining and content analyses of TTF revealed that the germinated seeds treated with GA_3_ had a significantly higher TTF contents than water-treated and dormant seeds (Fig. 1c), which indicated that GA_3_ treatment increases seed vigor.

To clarify the physiological mechanism by which GA_3_ promotes seed germination, we measured the water absorption rate of seeds among the CK, W, and G groups. The water absorption rate of seeds in the G group was significantly higher than those of seeds in the W and CK groups before saturation (within 8 h). The G seed group also revealed the highest water absorption rate (Fig. 1d). Thus, GA_3_-treated seeds germinated faster than the W and CK seed because the former absorbed more water during germination. Overall, GA_3_ treatment shortened the physiological dormancy period, improved seed vigor, and promoted seed germination in *F. hupehensis*.

### RNA-seq and de novo assembly of *F. hupehensis* reference transcriptome

Nine cDNA libraries (CK1, CK2, CK3, W1, W2, W3, G1, G2, and G3) were sequenced by Illumina HiSeq to detect the transcriptome level of gene expression information during *F. hupehensis* seed germination. Sequencing readings were deposited at the National Center for Biotechnology Information (NCBI) under SRA accession number SUB4314435. High-quality, clean reads were obtained by filtering low-quality reads obtained by sequencing. The Q30 of nine samples exceeded 94%, and the base content was uniform (Additional file 1: Table S1). A total of 116,932 unigenes were obtained after de novo assembly using Trinity software. The average length of unigenes was 82,979 bp, and the length of N50 was 1346 bp. The length of 68,984 (58.83%) unigenes were over 400 bp, while those of 30,024 (25.67%) unigenes were over 1000 bp (Table 1). Thus, the assembly quality of the transcriptome was satisfactory.

**Table 1:** Statistics of unigene stitching results

length range	Count	Percentage (%)
200–400	48,138	41.17
400–600	19,798	58.83
600–1000	11,388	16.23
1000–2000	19,358	16.55
2000–3000	6731	5.76
3000–4000	2547	2.18
4000+	1384	1.18
Total	116,932	
Length of N50	1690	
Average length	1081	

### Function annotation and classification of *F. hupehensis* unigenes

Trinotate was used to compare the sample unigene sequences with a common functional database (Table 2); here, 68,715 (58.765%) annotated unigenes were obtained from NR database; 41,776 (35.727%) were obtained from NT database; and 49,676 (42.483%), 33,484 (28.635%), 30,293 (25.907%), 47,270 (40.425%), and 23,771 (20.329%) were obtained from Swiss-Prot, PFAM, Cluster of Orthologous Group (COG), Gene Ontology (GO), and Kyoto Encyclopedia of Genes and Genomes (KEGG) databases, respectively.

**Table 2:** Functional annotations of unigenes in the NR, NT, Swiss-Prot, PFAM, COG, GO and KEGG databases

Database	NR	NT	Swiss-Prot	PFAM	COG	GO	KEGG
Count	68,715	41,776	49,676	33,484	30,293	47,270	23,771
Percentage	58.765%	35.727%	42.483%	28.635%	25.907%	40.425%	20.329%

**Fig. 2:**
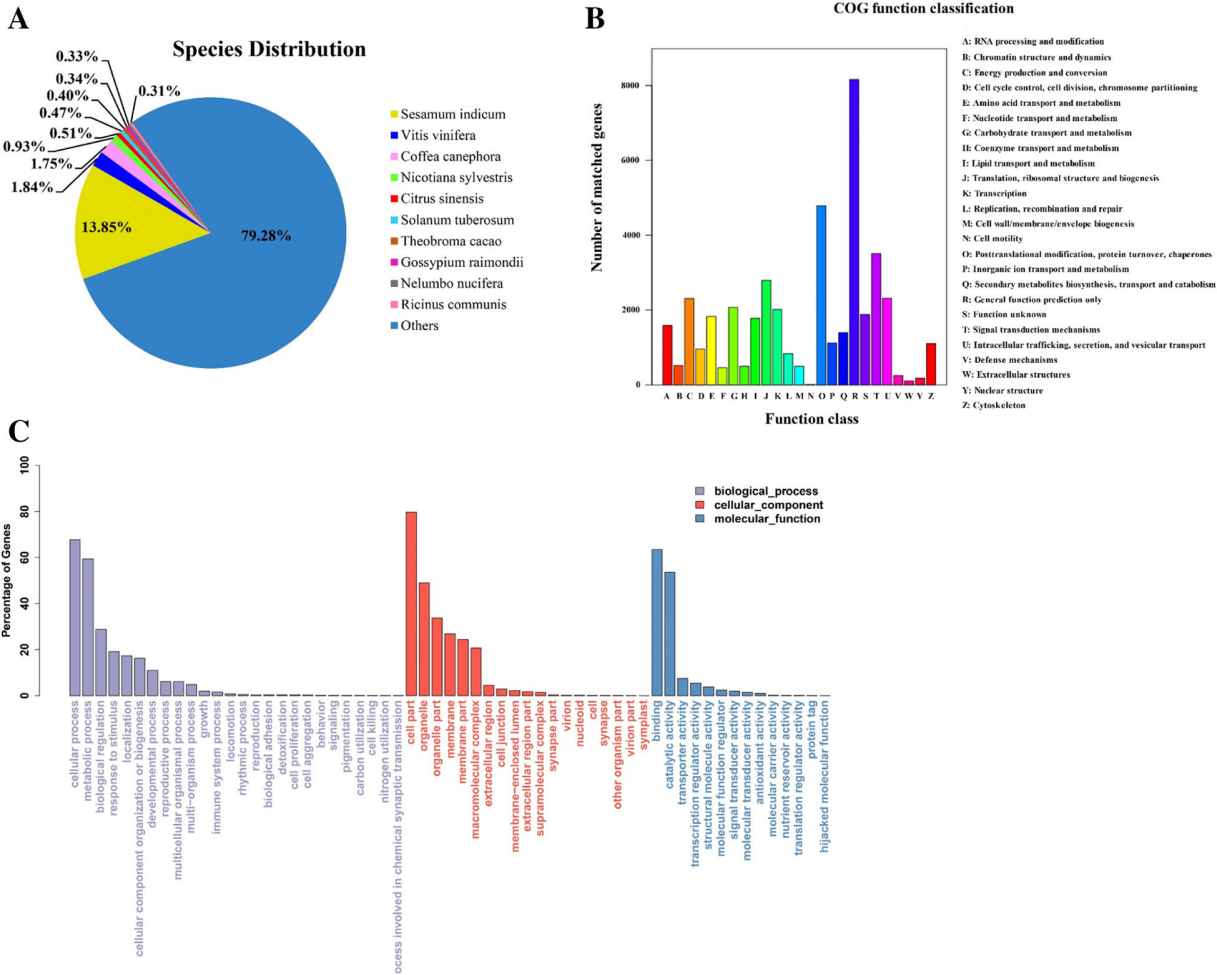
Functional annotations of the unigenes of the *F. hupehensis* seed transcriptome. **a** NR annotated species distribution map similar to the *F. hupehensis* transcriptome. **b** KOG function annotation of *F. hupehensis* seeds. **c** GO function annotation of *F. hupehensis* seeds

Nr is the non-redundant NCBI collection of nucleotide and protein sequence database. A total of 68,715 unigenes were annotated to the NR database (Fig. 2a). *F. hupehensis* transcripts were highly similar to those *Sesamum indicum* (13.85%), *Vitis vinifera* (1.84%), and *Coffea canephora* (1.75%).

COG is a database of orthologous gene families. A total of 30,293 unigenes of *F. hupehensis* were annotated to 25 COG pathways (Fig. 2b). A total of 8157 (19.01%) unigenes were annotated to the general function prediction classification, followed by 4776 (11.13%), 3507 (8.17%), 2790 (6.50%), 2009 (4.68%), and 834 (1.94%) annotations related to gene expression, including post-translational modification; protein turnover and chaperones; signal transduction mechanisms; translation, ribosomal structure, and biogenesis; and transcription, replication, recombination, and repair, respectively.

GO is a gene function database. A total of 47,270 unigenes from *F. hupehensis* were annotated into 59 GO pathways (Fig. 2c). The functions of unigenes in biological process classifications contained cellular process (67.76%), metabolic process (59.43%), and biological regulation (28.76%). Cell part (79.75%), organelle (49.04%), and organelle part (33.66%) were the most abundant functions in terms of cellular component classifications. In the molecular function classification, binding (63.47%), catalytic activity (53.55%), and transporter activity (7.49%) were more abundant. The main GO entries revealed that the cells divided frequently during seed germination, and some catalytic, metabolic, and binding activities were relatively high.

### Comparative analysis of DEGs

**Fig. 3:**
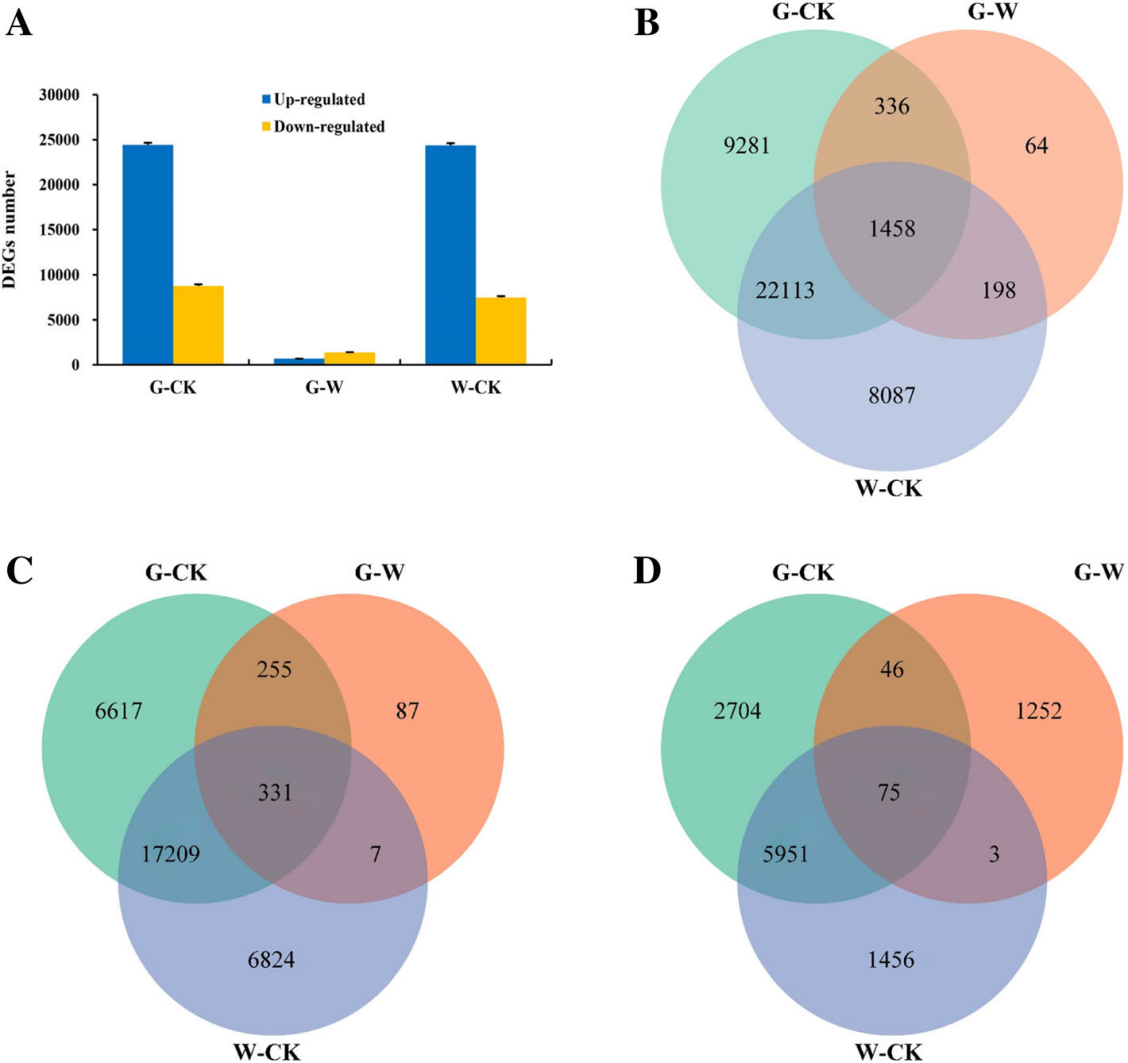
Statistical analysis of differentially expressed unigenes (DEGs) during *F. hupehensis* seed germination. **a** Statistical analysis of up/down-regulated unigenes in the CK, W, and G groups. **b** Venn diagram of all DEGs. **c** Venn diagram of up-regulated genes. **d** Venn diagram of down-regulated genes

DEGs were analyzed was performed using the RPKM method to determine the degree of overlap between the three seed groups. Compared with the dormant seeds, the total numbers of up-regulated genes in the germinated seeds treated with water and GA_3_ were 24,371 and 24,412, respectively. The numbers of down-regulated genes in the W and G groups were 7485 and 8776, respectively. Compared with the W seeds, G seeds contained 680 up-regulated genes and 1376 down-regulated genes (Fig. 3a, Additional file 2: Table S2). Various process genes were compared using Venn diagrams. A total of 31,856 DEGs were obtained between the W and CK groups, 33,188 DEGs were obtained between the G and CK groups, and 2056 DEGs were obtained between the G and W groups. A total of 1458 DEGs (331 commonly up-regulated and 75 commonly down-regulated) were shared among the three treatments (Fig. 3b-d, Additional file 2: Table S2), thus implying that these 1458 DEGs might be responsible for *F. hupehensis* seed germination. Correlation heat map analysis of the expression among samples revealed that the results were reproducible (Additional file 3: Figure S1). Based on similarities in gene expression, DEGs in the samples were generated by hierarchical clustering combined with K-means clustering. Hierarchical clustering of the gene expression profiles of the CK, W, and G seed groups showed that the DEGs could be divided into eight clusters (Additional file 4: Figure S2), and the genes of the same subclass had similar expression patterns (Additional file 2: Table S2, Additional file 5: Figure S3).

### KEGG pathway analysis of DEGs

**Fig. 4:**
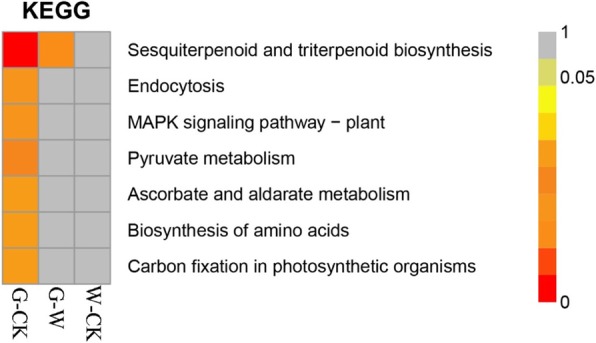
Analysis of KEGG enrichment of DEGs in *F. hupehensis*

KEGG is a signal pathway database with an extremely rich signal pathway map, and the map of interaction between genes contained in a pathway. Enrichment of DEGs in the KEGG pathway was analyzed at a significance level of *p* < 0.05. The KEGG annotations indicated that 136 pathways between the G–CK groups and W–CK and 108 pathways between the G and W groups were enriched. In particular, the sesquiterpenoid and triterpenoid biosynthesis (map 00909), endocytosis (map 04144), MAPK signaling pathway-plant (map 04016), pyruvate metabolism (map 00620), ascorbate and aldarate metabolism (map 00053), biosynthesis of amino acids (map 01230), and carbon fixation in photosynthetic organisms (map 00710) pathways between the G and CK groups were significantly enriched. DEGs were significantly enriched in the sesquiterpenoid and triterpenoid biosynthesis (map 00909) pathways between the G and W groups. No significant difference in pathway was observed between the W and CK groups (Fig. 4). Some terpenoids, such as gibberellin, abscisic acid, and other plant hormones, are necessary for plant growth and development, whereas some species were necessary in regulating the relationship between plant and environment [23].

### DEGs related to sugar metabolism in seed germination

**Fig. 5:**
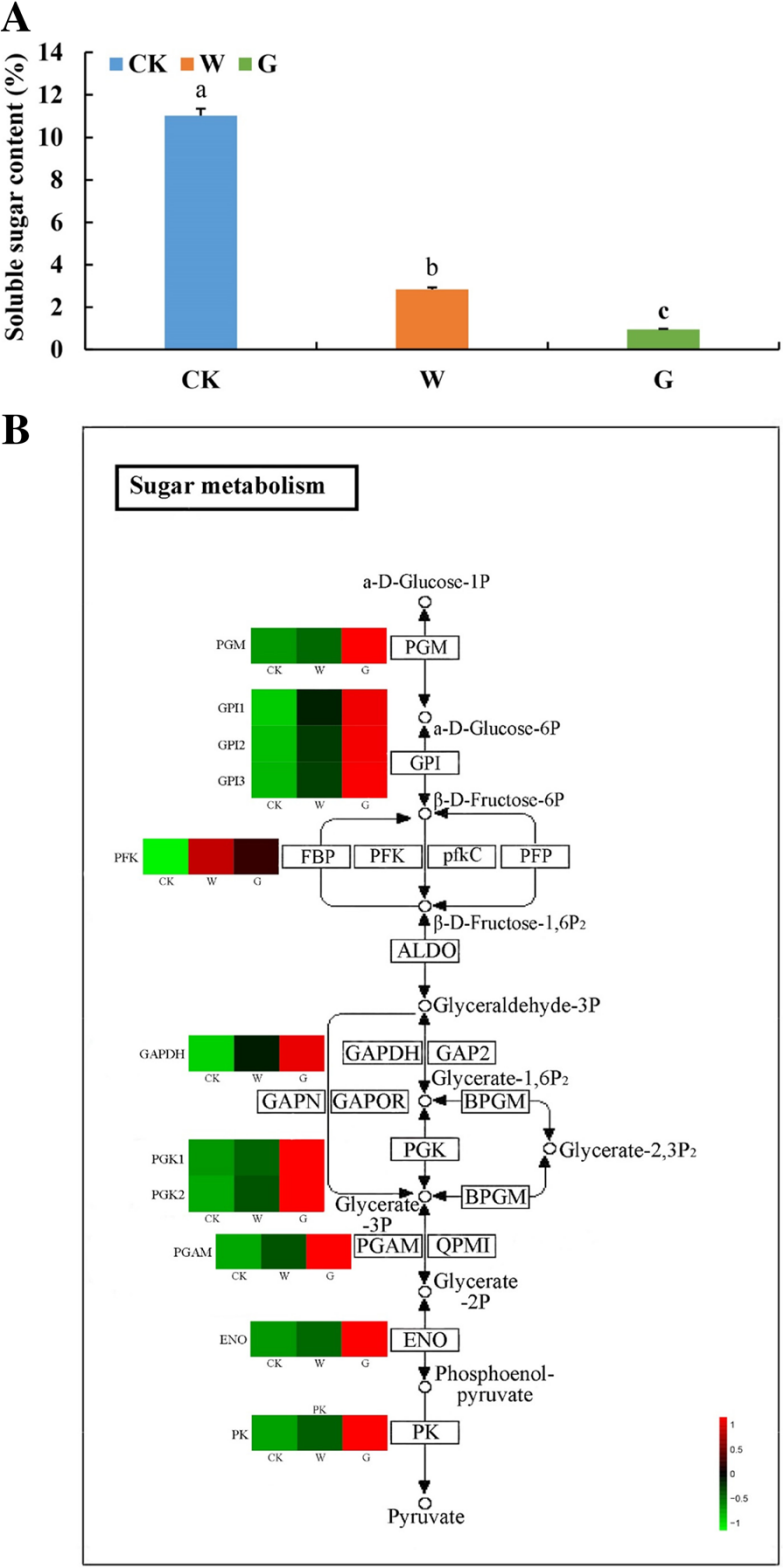
Sugar content and expression profiles of DEGs related to sugar metabolism during *F. hupehensis* seed germination. **a** Comparison of sugar content in *F. hupehensis* seed under three treatments. **b** Expression profiles of DEGs related to sugar metabolism in *F. hupehensis* seed under three treatments. The sample names are indicated at the bottom of the figure. Changes in expression level are represented by a change in color; green indicates a lower expression level, whereas red indicates a higher expression level. All data shown indicate the average mean of three biological replicates (*n* = 3). Means with different letters in each treatment represent a significant difference at *p* ≤ 0.05

The highest content of soluble sugar was found in dormant seeds, followed by the W seeds, and finally G seeds (Fig. 5a). The KEGG annotations suggested that eleven DEGs were related to the sugar pathway in *F. hupehensis* seeds. Cluster analysis revealed that the expression levels of all eleven DEGs significantly differs among the three seed groups (Fig. 5b). Phosphoglycerate kinase (*PGK*), phosphoglucomutase (*PGM*), pyruvate kinase (*PK*), enolase (*ENO*), 2,3-bisphosphoglycerate-dependent phosphoglycerate mutase (*PGAM*), glyceraldehyde 3-phosphate dehydrogenase (*GAPDH*), glucose-6-phosphate isomerase (*GPI*), and 6-phosphofructo-2-kinase (*PFK*) were more highly expressed in germinated seeds was higher than that in dormant seeds, especially in G group seeds (Additional file 6: Table S3). The expression levels of DEGs were consistent with the trends of soluble sugar content, thereby indicating that these eleven DEGs might play important roles in promoting seed germination. Hence, exogenous GA_3_ could promote the expression level of key genes in the sugar metabolic pathway of *F. hupehensis* seeds.

### Hormone concentrations and hormone-related DEGs in seed germination

**Fig. 6:**
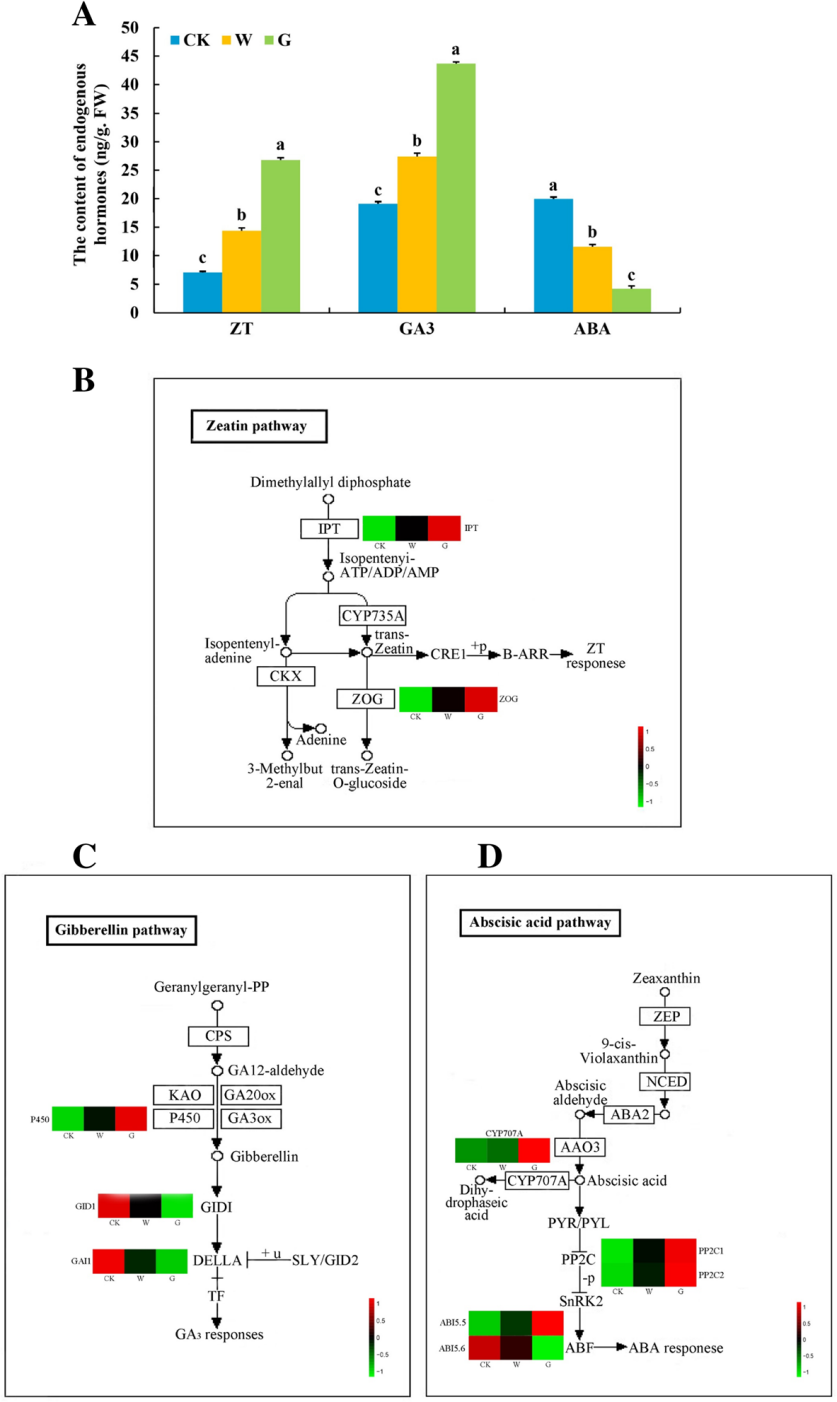
Endogenous ZT, GA_3_, and ABA concentrations and expression profiles of DEGs related to hormone pathways involved in *F. hupehensis* seed germination. **a** Comparison of ZT, GA_3_, and ABA concentrations in *F. hupehensis* seeds under three treatments. **b** Expression profiles of DEGs related to the ZT pathway in *F. hupehensis* seeds under three treatments. **c** Expression profiles of DEGs related to the GA_3_ pathway in *F. hupehensis* seeds under three treatments. **d** Expression profiles of DEGs related to the ABA pathway in *F. hupehensis* seeds under three treatments. The sample names are shown at the bottom of the figure. Changes in expression level are indicated by a change in color; green indicates a lower expression level, whereas red indicates a higher expression level. All data shown reflect the average mean of three biological replicates (*n* = 3). Means with different letters in each treatment represent a significant difference of *p* ≤ 0.05

To investigate the roles of endogenous hormones in seed germination, we determined the ZT, GA_3_, and ABA contents in *F. hupehensis* seeds. Both ZT and GA_3_ contents were significantly the highest, but ABA content was significantly the lowest in the G group. By contrast, both ZT and GA_3_ contents were significantly the lowest whereas ABA content was significantly the highest in the CK group (Fig. 6a). Therefore, GA_3_ treatment could promote seed germination through endogenous hormone accumulation. Furthermore, exogenous GA_3_ induced seed germination by increasing ZT and GA_3_ concentrations and decreasing ABA concentration, thus conforming to the transcriptome data. Pairwise comparison of the treatments (W–CK, G–CK, and G–W) revealed that two DEGs were related to ZT biosynthesis in *F. hupehensis*. Cluster analysis of genes revealed that the expression levels significantly differs among the three seed groups (Fig. 6b). The expression levels of adenylate isopentenyltransferase (*IPT*) and zeatin O-glucosyltransferase (*ZOG*) were higher in germinated seeds than that in dormant seeds, especially in the G group seeds, (Additional file 6: Table S3). These results were in accordance with those of endogenous hormone ZT content and indicated that the identified DEGs had a vital function in accelerating *F. hupehensis* seed germination. These results revealed that exogenous GA_3_ treatment could advance ZT biosynthesis by regulating the key genes.

From the *F. hupehensis* transcriptome, we obtained a key gene cytochrome P450 (*CYP450*) in the GA biosynthetic pathway, *CYP450* was significantly highly expressed in the G seeds, followed by W seeds, and least expressed in dormant seeds (Fig. 6c, Additional file 6: Table S3). Moreover, cluster analysis revealed that the expression levels of two DEGs associated with the GA_3_ signal transduction pathway significantly differed among the three seed groups (Fig. 6c). The expression levels of *GAI1* and gibberellin receptor (*GID1*) were significantly lower in the G group than in the W group and lowest in the CK group (Additional file 6: Table S3). Thus, the expression levels of *GAI1* and *GID1* were down-regulated by GA_3_ treatment, thereby increasing the hormonal signal transduction of GA_3_ during *F. hupehensis* seed germination.

One DEG was related to the ABA biosynthetic pathway and three DEGs in the ABA signal transduction pathway were observed. The expression levels of four DEGs significantly differs among the three groups (Fig. 6d). (+)-Abscisic acid 8′-hydroxylase (*CYP707A*), ABSCISIC ACID-INSENSITIVE 5 like 5 (*ABI5.5*), and protein phosphatase 2C (*PP2C*) were highly expressed in the G group but least expressed in the CK group. By contrast, the expression level of ABSCISIC ACID-INSENSITIVE 5 like 6 (*ABI5.6*) in dormant seeds was significantly higher than that in germinated seeds, and its expression level in GA_3_-treated seeds was significantly higher than that in water- treated seeds (Additional file 6: Table S3). Thus, exogenous GA_3_ may be conducive to alleviate the inhibitory effect of ABA signal transduction on the seed germination of *F. hupehensis*.

### Other important DEGs related to seed germination

We examined several impartment pathways involved in seed germination. KEGG pathway analysis revealed that one DEG (pyruvate dehydrogenase E1 component beta subunit) related to the TCA cycle was clustered, and its highest and lowest expressions were observed germinated seeds (G group) and the CK group, respectively (Additional file 6: Table S3). Moreover, one DEG (auxin-responsive GH3 gene family) related to the auxin signaling pathway was expressed the highest in the germinated seeds (G group) and the least in the CK group (Additional file 6: Table S3). Seven DEGs were related to antioxidants, and the expression levels of catalase (*CAT*) and glutathione S-transferase (*GST*) were significantly higher in the G group than in the W group but lowest in the CK group. Moreover, superoxide dismutase (*SOD*), peroxiredoxin (*POD*), and glutamate-cysteine ligase (*GCL*) were expressed the highest in the germinated seeds (W group) but the least in the CK group (Additional file 6: Table S3). Twelve DEGs were associated with mRNA degradation. Among these 12 DEGs, the expression levels of seven subunits of CCR4-nontranscriptional complex (*CCR4-NOT*), enhancer of mRNA-decapping protein (*Edcp*), and 5′-3′exoribonuclease (*XRN2*) were significantly greater in the W group than in the G group but lowest in the CK group. Meanwhile, the expression level of U6 snRNA-associated Sm-like protein LSm4 was highest in the G group (Additional file 6: Table S3).

### DEGs related to TFs in seed germination

**Fig. 7:**
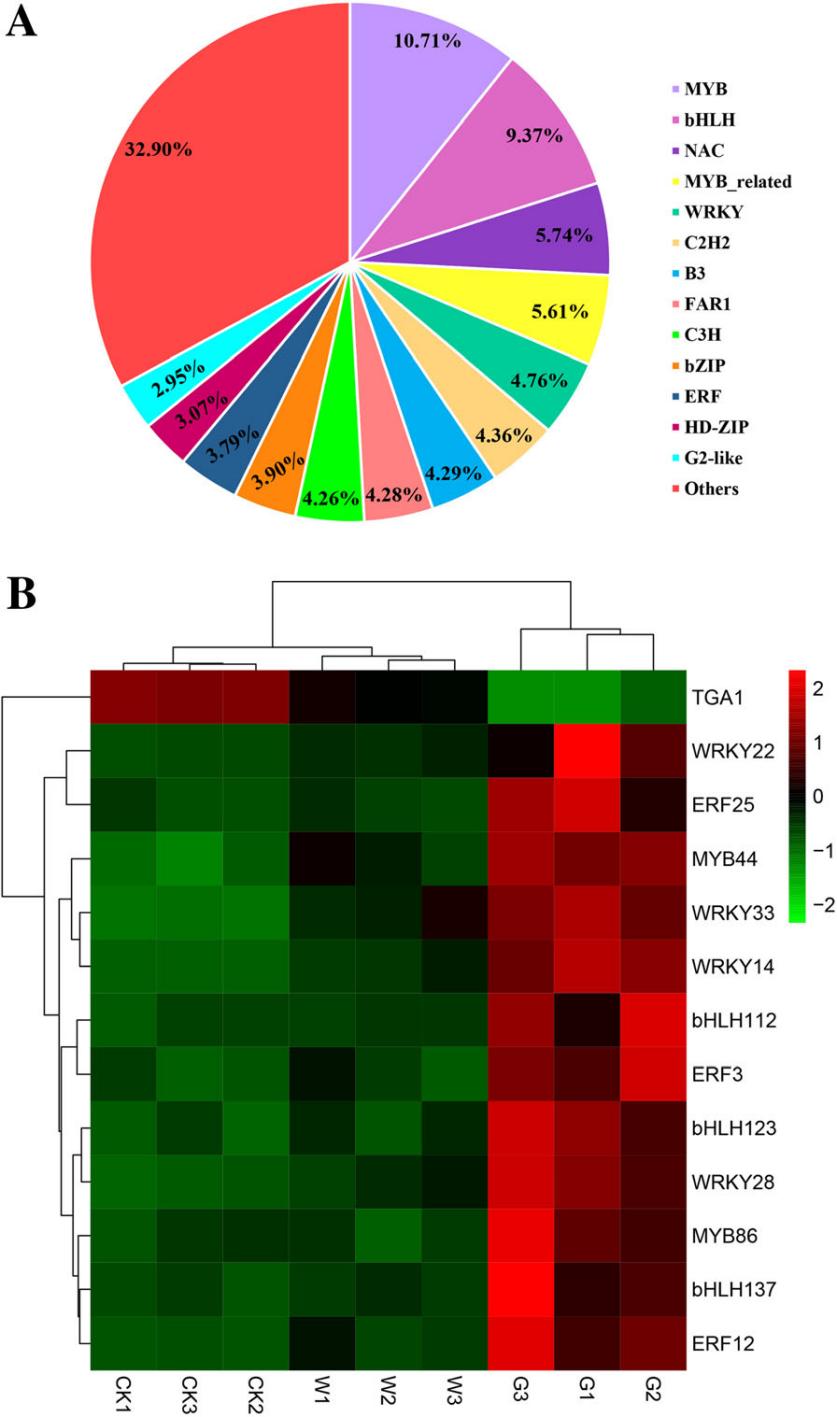
Analysis of transcription factors (TFs) involved in *F. hupehensis* seed germination. **a** Distribution of TF families in the transcriptome of *F. hupehensis* seeds. **b** Expression profiles of differentially expressed TFs involved in *F. hupehensis* seed germination. The sample names are shown at the bottom of the figure. Changes in expression level are represented by a change in color; green indicates a lower expression level, whereas red indicates a higher expression level. All data shown reflect the average mean of three biological replicates (*n* = 3). Means with different letters in each treatment represent a significant difference at *p* ≤ 0.05

The gene expression network regulated by TFs plays an important role in the growth and development of plants [24]. A total of 155,244 TFs were annotated in the transcriptome of *F. hupehensis* seeds and classified into 56 families (Fig. 7a). To better understand the molecular mechanism underlying the effects of GA_3_ treatment on seed germination, we measured the differential expression of TFs by cluster analysis and obtained 13 TFs with significant differences from 5 TF families among the three seed groups (Fig. 7b). Pairwise comparison (W–CK, G–CK, and G–W) showed that significantly up-regulated TFs included two TF members belonging to the MYB family (MYB44 and MYB86), four TFs from the WRKY family (WRKY14, WRKY22, WRKY28, and WRKY33), three TFs from the ERF family (ERF3, ERF12, and ERF25), and three TFs from the bHLH family (bHLH112, bHLH123, and bHLH137). The significantly down-regulated TFs included one TGA1 belonging to the bZIP family (Additional file 7: Table S4). These TFs might play significant roles in expediting the seed germination of *F. hupehensis*. Moreover, the mechanism by which GA_3_ regulates *F. hupehensis* seed germination involved an extremely intricate and complex transcriptional network. These findings provided basic information for studying the role of TFs in the promoting effect of GA_3_ on the seed germination of *F. hupehensis*.

### Validation of DEGs by qRT-PCR

**Fig. 8:**
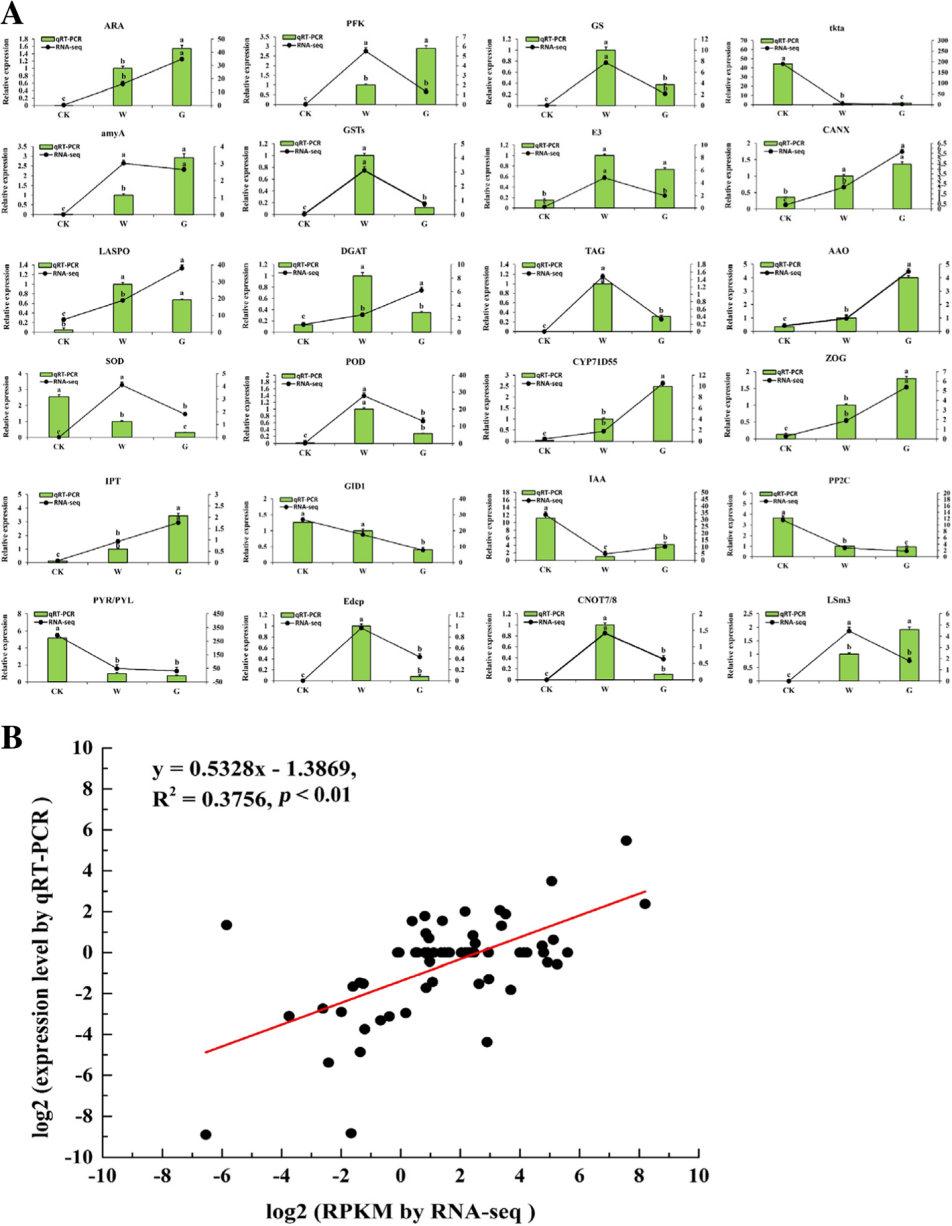
qRT-PCR verification diagram of DEGs during *F. hupehensis* seed germination. **a** Comparison of the expression levels determined by qRT-PCR and RNA-seq from three treated seeds. **b** Correlation plot of the RNA-Seq results (RPKM) and qPCR (2^-ΔΔCt^) results. Result were calculated using log2 fold variation measurements. The *R*^2^ value represents the correlation between the RNA-seq and qPCR results. All data indacate mean ± SE (*n* = 3 with 500 seeds per replicate). Means with different letters in each treatment representa significant difference at *p* ≤ 0.05

To verify the accuracy and reproducibility of the RNA-seq results, we randomly selected 24 genes in the related pathways of seed germination for qRT-PCR validation (Additional file 8: Table S5). The expression levels of the selected genes were calculated using the 2^-ΔΔCt^ method. We compared the expression data of the three groups obtained by RNA-seq and qRT-PCR (Fig. 8a). The correlation between RNA-Seq results (RPKM) and qPCR results (2^-ΔΔCt^) results for the 24 DEGs was calculated using log_2_ fold variation measurements to produce a scatter plot. The qRT-PCR results of 24 DEGs were significantly similar to the RNA-seq results (*R*^*2*^ = 0.38, *p* < 0.01; Fig. 8b), which indicated that the RNA-seq data were reliable and accurate.

## Discussion

### *F. hupehensis* reference transcriptome

Seed germination is based on the prominence of the radicle, some criteria for judging seed germination are based on the fact that some biological processes during seed germination mainly include nutrient metabolism, transcription, DNA repair, cell elongation and cell division recovery [25, 26]. Therefore, detailed information on gene expression is critical to understand the molecular mechanisms of any developmental process. An increasing number of analytical tools are being used to study the mechanism of seed dormancy, germination, and development [13, 19, 27]. Although *F. hupehensis* has great economic value, complete gene information for this species remains unavailable. In this current study, we describe the gene expression profiling of *F. hupehensis* seed germination under GA_3_ treatment. This work represented the first attempt to use Illumina sequencing technology to further understand the transcriptome of *F. hupehensis* seeds. RNA-seq was performed using Illumina HiSeq sequencing, which assembled 116,932 unigenes. The unigenes were used to perform BLASTX-based searches and annotations on the NT, NR, Swiss-Port, PFAM, GO, COG, and KEGG databases. Several key pathways associated with seed germination were also obtained by KEGG annotation analysis to identify DEGs in these pathways based on RPKM values combined with qRT-PCR data. Our transcriptome data suggested that a large number of DEGs were involved in various metabolic pathways, most of which were related to the regulation of gene expression, followed by energy production and metastasis. Only a small fraction of the DEGs found were related to signal hormone transduction and reactive cell division.

### Role of sugar in *F. hupehensis* seed germination

**Fig. 9:**
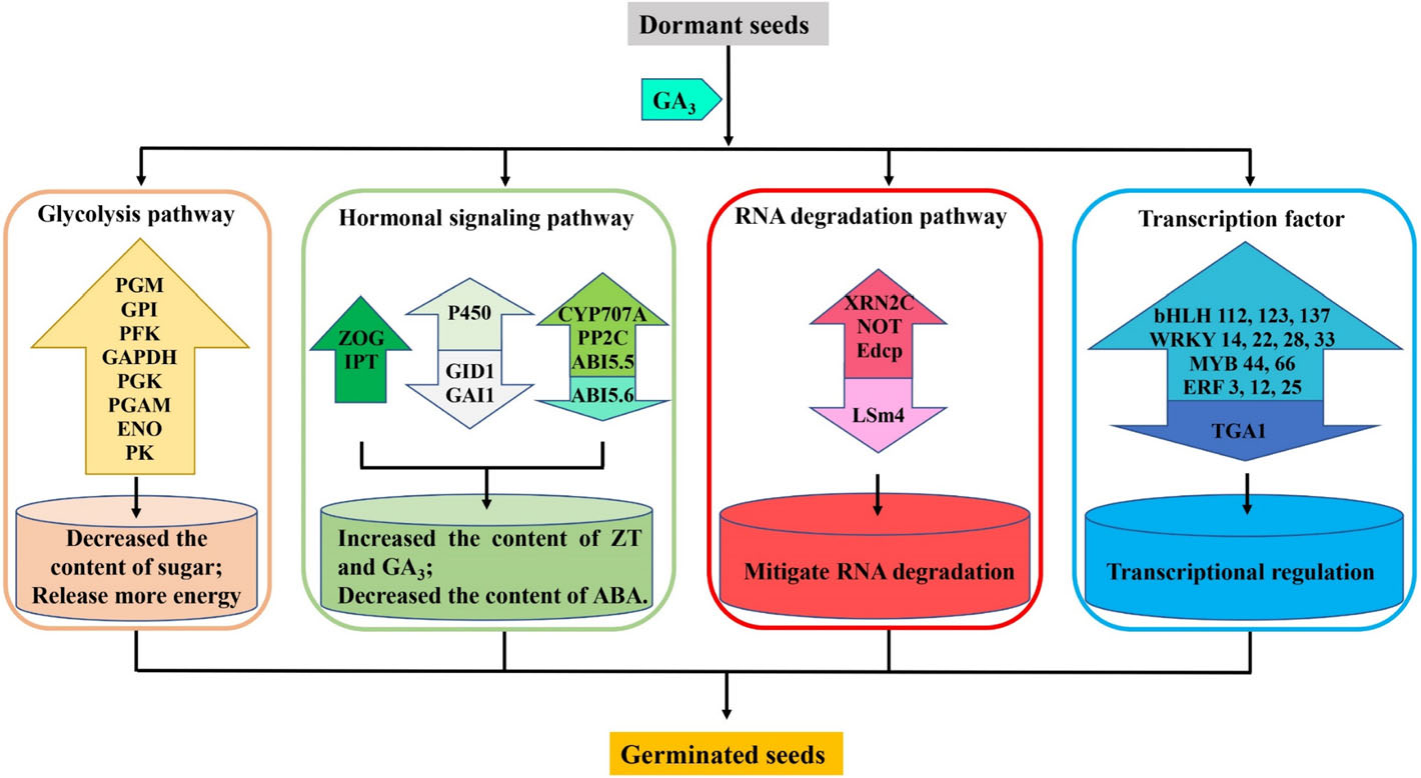
Regulation model of exogenous GA_3_ and cold treatment promoting *F. hupehensis* seed germination. The colored arrow pointing upward indicates the up-regulation of genes, whereas the colored arrow pointing downward indicates the down-regulation of genes

During seed germination, soluble sugars are mostly used to synthesize or transform other substances, thus providing energy for seed germination and seedling growth [28]. Exogenous GA_3_ treatment can reduce the sugar contents [29, 30], which was consistent with our measurements of the lowest sugar content in germinated seeds treated with GA_3_. In addition, eleven DEGs (*PGM*, three *GPIs*, *PFK*, *GAPDH*, two *PGKs*, *PGAM*, *ENO*, and *PK*) involved in the glycolytic pathway were identified from the RNA-seq data, which implied that exogenous GA_3_ up-regulated these key genes to convert a-D-glucose-1P into pyruvate, which is the final product of the glycolytic pathway and is used for the metabolic conversion of intermediates in other substances (Fig. 9). Germinating seeds require more nutrients, and exogenous GA_3_ promoted seed germination by accelerating the oxidative decomposition of sugars to affect their mobilization of sugar and up-regulating the expression of key genes in the glycolytic pathway in a consistent direction to provide energy for *F. hupehensis* seed germination.

### Energy requirements for *F. hupehensis* seed germination

A considerable amount of energy is required during seed germination, and all biochemical reactions in plants enter the TCA cycle, providing power for the mitochondrial electron transport chain to generate ATP [31]. The energy required during seed germination mainly comes from glycolysis, pentose phosphate pathway, and the TCA cycle of cellular respiration [32]. The ATP content in dry seeds is low but gradually increases after seed germination, which proves that the respiratory pathway plays an important role in seed germination [33, 34]. The degradation products of energy reserve always undergo glycolysis, after which ATP synthesis is synthesized through the TCA cycle and mitochondrial electron transport. In the present study, KEGG annotation analysis suggested that eight genes (*PGM*, *GPI*, *PFK*, *GAPDH*, *PGK*, *PGAM*, *ENO*, and *PK*) related to the glycolytic pathway were hardly expressed in the dormant seeds but highly expressed in germinated seeds, especially in the GA_3_-treated seeds (Fig. 9). Hence, GA_3_ and water treatment required large amounts of energy to germinate seeds, and the energy requirement for most seed germination events was primarily achieved by glycolysis [10]. However, when the energy provided by anaerobic respiration cannot satisfy the needs of germinating seeds, the seeds undergo the TCA cycle to provide considerable energy for germination under oxygen-rich conditions [18]. In this study, KEGG metabolic pathway analysis identified *PDHB* as a group of rate-limiting enzymes in the TCA cycle that catalyzed the irreversible oxidative decarboxylation of pyruvate to acetyl-CoA; those enzymes connected the aerobic oxidation of sugars with the TCA cycle, thereby playing an important role in the energy metabolism of the mitochondrial respiratory chain [35]. The expression level of *PDHB* significantly differed among the three groups, with the highest expression found in the germinated seeds (especially in the G group) and the lowest expression found in the dormant group. Our results were consistent with the results of Weitbrecht et al. [31], who demonstrated that the pyruvate dehydrogenase transcript accumulates during cold stratification and GA_3_ treatment. Thus, although both water and GA_3_ treatments promoted seed germination and required more energy for seed germination, exogenous GA_3_ accelerated the TCA cycle to release more energy for seed germination by up-regulating *PDHB*. These respiratory pathways are critical for providing energy for various cellular functions during seed germination, as described in our study [32, 36]. Most of this energy was produced during germination, especially after GA_3_ treatment. Thus, *F. hupehensis* seeds must consume more sugar for energy conversion during germination.

### Comparison of ZT, GA_3_, and ABA involved in *F. hupehensis* seed germination

ZT is the first type of cytokinin isolated and identified in plant growth and development; it is mainly present in seeds with high metabolic metabolism and presents antagonistic effects on seed germination inhibitors [37]. ZT was discovered in the hypocotyl of chickpea and found to promote nutrient storage and metabolism in cotyledons [38]. Pea seeds have peak ZT contents during germination, especially during radicle prominence [39]. Similarly, ZT contents in *F. hupehensis* seeds were highest after treatment with GA_3_, followed by that after treatment with water, and then in dormant seeds. These results suggest that ZT promoted the protrusion and extension of seed radicle. When plant needs ZT, ZT is formed by removing the nitrogen chain of ZT and then modifying to form ZT again [31]. In the present study, the expression level of IPT, which is the first key enzyme in the biosynthetic pathway of ZT, was significantly higher in *F. hupehensis* seeds germinated by GA_3_ treatment than in those subjected to other treatments. Trans-ZT was synthesized by cytokinin trans-hydroxylase (CYP735A) [40], which was also highly expressed in the germinated seeds treated with GA_3_. ZOG is an important enzyme in the trans-ZT metabolic pathway [41, 42]. Our data showed that the expression level of *ZOG* was significantly higher in the germinated seeds treated with GA_3_ than in those treated with water, indicating that the anabolism of ZT was relatively strong under GA_3_ treatment (Fig. 9). This result might be related to ZT mobilization of reserve metabolism and promotion of radicle protrusion [39]. Taken together, GA_3_ treatment could accelerate the anabolism of ZT to facilitate seed germination in *F. hupehensis*.

GA_3_ regulates various developmental processes, including seed germination and seedling development, throughout the plant life cycle [14, 39]. GA_3_ can break through the mechanical constraints of seed coats during seed germination to promote radicle protrusion [14, 43]. In addition, GA_3_ promotes cell division, which is vigorous during seed germination and related to the synthesis and catabolism of GA_3_ [44]. Consistent with the results of previous studies, our data showed that the GA_3_ content was the highest in *F. hupehensis* seeds treated with GA_3_. GA_3_ is biosynthesized from geranylgeranyl-PP, which is converted into GA_12_ by ent-copalyl diphosphate synthase. GA_12_ is then converted into GA_3_ by KAO, CYP450, GA_20_ox, and GA_3_ox enzymes [45]. In the present study, GA_3_ treatment significantly up-regulated the expression of the *CYP450* gene in the GA_3_ biosynthetic pathway of *F. hupehensis* seeds. Some key genes in the GA_3_ signaling pathway also determined seed germination. DELLA protein is a plant growth inhibitor, while GID1 is a receptor for GA_3_ that degrades DELLA protein in plants by binding to GID1 receptors [46, 47]. GA_3_ expedites germination by down-regulating some germinated inhibitory proteins, such as DELLA [15, 48]. In the present work, *GAI1* and *GID1* expression levels were significantly lower in the GA_3_-treated seeds than in the water-treated seeds, and highest in dormant seeds. Overall, although both water and GA_3_ treatment could regulate the expression of related genes in GA_3_ pathway, exogenous GA_3_ could significantly up-regulate genes in GA_3_ biosynthesis and down-regulate negative regulators in GA_3_ signal transduction pathways. These phenomena increased the accumulation levels of GA_3_ and contribute to seed germination; this supposition was supported by the observed increase in endogenous GA_3_ concentration in *F. hupehensis* seeds (Fig. 9).

ABA regulates the accumulation of phytochemicals in seeds and controls the dehydration of seed development at later stages [49]. ABA maintains a relatively high level in dry seeds and rapidly declined after germination [50, 51]. Moreover, the adequate reduction of endogenous ABA content is a major prerequisite for complete germination because ABA inhibits the weakening and rupture of endosperm [52, 53]. GA_3_ can promote seed germination and counteract ABA inhibition in seeds [54]. Similar results were obtained in the present experiments, in which ABA contents were highest in dormant seeds and lowest in fast-germinating seeds (GA_3_-treated germinated seeds). ABA is synthesized in the carotenoid pathway, starting with zeaxanthin. NCED rapidly catalyzes the synthesis of ABA when seeds absorber water and is a key enzyme for ABA synthesis [55], whereas CYP707A is the core enzyme of ABA degradation [56]. The expression levels of ABA metabolic genes are associated with inhibition of seed germination [20, 32]. In the current study, the expression level of *NCED* in the dormant seeds of *F. hupehensis* was significantly higher than that in germinated seeds, but no significant difference was observed in the seeds after germination. This result suggested that the amount of ABA synthesized was relatively small during the germination. By contrast, *CYP707A* expression in the GA_3_-treated seeds was significantly higher than that in other seeds, which indicated that *CYP707A* mainly degraded ABA content in *F. hupehensis* seeds during germination. Our results revealed that water and GA_3_ treatment accelerated the full degradation of ABA content, but GA_3_ treatment was effectively increased the expression of the degrading gene *CYP707A* and resisted the inhibitory effect of ABA on seed germination. In the signaling pathway, *PP2C* is negatively correlated with the regulation of the ABA signaling pathway in spinach and *Arabidopsis* seeds [57, 58]. In the present work, *PP2C1* and *PP2C2* were highly expressed in germinated seeds treated with GA_3_ but minimally expressed in dormant seeds. ABI5 is a member of the basic leucine zipper (bZIP) family, which mediates cell responses to ABA in seeds and vegetative tissues [59]. The *ABI5* gene negatively regulates seed germination in *Arabidopsis thaliana* [60–62]. Our RNA-seq results revealed that the expression of *ABI5.6* in dormant seeds was significantly higher than that in germinated seeds, thus further verifying the role of *ABI5* in seed germination. Interestingly, the expression level of *ABI5.5*, another member of the ABI5 family, contrasted that of *ABI5.6*, which was also inconsistent with previous studies. This contradiction might be attributed to differences in ABI5 family members or differences among species. The role of these two members of the ABI5 family in the seed germination of *F. hupehensis* requires further verification. The results implied that GA_3_ significantly increased the expression of negative regulatory genes in the ABA signaling pathway and inhibited the effect of ABA on seed germination. In summary, exogenous GA_3_ reduced the ABA content of seed by degradation of gene related to ABA pathway and then significantly up-regulated the negative feedback factors in the ABA signaling pathways to promote the seed germination in *F. hupehensis* (Fig. 9). The decrease in endogenous ABA during seed germination also supported this view.

### Candidate TFs associated with *F. hupehensis* seed germination

In the present study, 155,244 TFs were obtained and classified into 56 families from the *F. hupehensis* transcriptome. Among these TF families, MYB, bHLH, WRKY, C2H2, FAR1, C3H, bZIP, and ERF accounted for a relatively large proportion. Our results were basically similar to those of the Tibetan *Sophora moorcroftiana* [63], *Lepidium apetalum* [64] and Chrysanthemum [65] transcriptomes, in which NAC, MYB_related, WRKY, C2H2, B3, FAR1, C3H, bZIP, ERF, and HD-ZIP TFs exert certain regulatory effects on plant growth and development and signal transduction [66].

The MYB family is the largest class of TFs in plants. Members of this family are mainly involved in plant growth and development and response to primary and secondary metabolic reactions [67]. In *Arabidopsis* seeds, the level of MYB44 transcript was up-regulated by 4 °C treatment as a negative regulator of ABA signaling [68]. Interestingly, MYB44 was also differentially up-regulated in germinated seeds after treatment with GA_3_ in *F. hupehensis*. We identified another MYB66 with the same up-regulated expression pattern as MYB44 in the G group of *F. hupehensis* seeds. Therefore, MYB might play an important regulatory role in seed germination with exogenous GA_3_ treatment. bHLH is the second largest TF class in plant seeds, and it plays an important role in regulating plant growth and development [69]. This TF class a light-stable repressor of seed germination and mediates the germination response to temperature [70]. Phytochrome-interacting factors, which are members of the *Arabidopsis* bHLH TF family, participate in light and gibberellin signal transduction in *Arabidopsis* and rice [71]. WRKY TFs are involved in various plant activities, such as development and metabolism [72], growth and senescence [73], and response to biotic and abiotic stresses [74]. Many proteins in the ERF family have been associated with different functions in cell processes, such as hormone signaling [75], regulation of metabolism [76], and developmental processes in various plant species [77]. In the present study, the expression levels of three bHLHs, four WRKYs, and three ERFs were significantly higher in the G group than in the two other groups of *F. hupehensis* seeds, thereby indicating that these 10 TFs might play some positive regulatory roles in the GA_3_ regulatory seed germination pathway (Fig. 9).

bZIP regulates various biological processes, such as pathogen defense, light and stress signals, seed maturation, and flower development [78]. bZIP16 is mainly expressed in seeds and could activate the GA pathway and inhibit ABA action, thereby promoting seed germination [79]. TGAla has been isolated from tobacco as a member of the bZIP class of TFs [80], whereas TGA1 and TGA4 regulated SA biosynthesis [81]. Comparison of our transcriptome data revealed that a TGA1 TF was a DEG among the different groups. This TF was highly expressed in dormant seeds but showed the lowest transcription level in GA_3_-treated seeds. Therefore, exogenous GA_3_ could play a negative role in the expression of TGA1, and the expression of TGA1 could inhibit inhibited seed germination (Fig. 9).

### Candidate genes related to RNA degradation associated with *F. hupehensis* seed germination

In eukaryotes, two main pathways of mRNA decay are triggered by the shortening of poly(A) of mRNA. In the 5′–3′ pathway, mRNA is degraded by 5′–3′ exonuclease [82], removed by an enhancer of mRNA-decapping protein [83], and required to accurately cleave the development of the U6 snRNA-associated Sm-like protein [84]. In the 3′–5′ pathway, the CCR4-NOT transcription complex subunit degrades mRNA and inhibits translation and transcription [85]. Degradation of mRNAs stored in seeds is a prerequisite for germination [86, 87], and these two pathways of RNA degradation contribute to seed germination [88]. In the present study, KEGG pathway analysis revealed that twelve DEGs were associated with mRNA degradation. As a result, CCR4-NOT was significantly highly expressed in germinated seeds and no transcripts were detected in dormant seeds, consistent with the findings above. The positive effects of GA_3_ treatment on seed hypocotyl growth are related to their effects on nucleic acid [89], and this treatment delays RNA degradation [90]. In the present study, similar results were found in the seeds of *F. hupehensis* transcriptome, that was, the expression level of seven subunits of the CCR4-NOT complex were significantly higher in water-treated seeds than that in GA_3_-treated seeds. Conversely, the expression level of U6 snRNA-associated Sm-like protein LSm4 was the highest in GA_3_-treated seeds, probably due to LSm4, which precisely cleaved the mRNA required for development. Thus, GA_3_ treatment leaded seeds to a more active germination state. In summary, increased in the gene expression of degraded RNA promoted the seed germination of *F. hupehensis*, GA_3_ could delay the degradation of RNA and induce *F. hupehensis* seeds to a favorable germination state for subsequent germination (Fig. 9).

## Conclusion

Exogenous GA_3_ increased seed vigor and water absorption rates to promote the seed germination of *F. hupehensis*. Transcriptomics approaches were used to study the effects of GA_3_ on *F. hupehensis* seed germination. A total of 116,932 unigenes were obtained from *F. hupehensis* seeds. Many DEGs involved in seed germination were obtained by comparing the transcriptomes of the seeds. Some key genes related to sugar, energy, hormones, RNA degradation, and some important TFs were involved in the seed germination of *F. hupehensis* (Fig. 9). The sugar metabolism level, the role of hormones, and the expression patterns of some important genes in pathways related to seed germination were further verified by measuring several physiological indicators and endogenous hormone contents, as well as qRT-PCR analysis. Differential expression analysis showed that GA_3_ also accelerated the oxidative decomposition of sugar by up-regulating key genes in the glycolytic pathway to release energy for germination. The expression levels of key genes related to hormone synthesis and signal transduction were affected by GA_3_ treatment, as well as the contents of three endogenous hormones (ZT, GA_3_, and ABA) in *F. hupehensis* seeds. GA_3_ could delay the RNA degradation of germination seeds to maintain a favorable germination state. In addition, some TF genes such as MYB, WRKY, ERF, bHLH, and bZIP were up-regulated by GA_3_, thus suggesting that these TFs played important roles in seed germination (Fig. 9). The transcriptome data of *F. hupehensis* can be used by further studies on low-fecundity and endangered species and provide theoretical evidence for protecting and utilizing these valuable resources.

## Methods

### Plant materials

Seeds were collected from a 25-year-old *F. hupehensis* and grown in Jingshan Botanical Garden (31°03′N, 113°11′E) in Yongxing Town, Jingshan County, Jingmen City, Hubei Province. Sampling of the *F. hupehensis* seeds was approved by Jingmen Forestry Bureau before collection. After the winged perianth was removed, the seeds were divided into three experimental treatments: dormant seed (CK) and germinated seeds subjected to low-temperature lamination treated with water (W) and GA_3_ (G). Each treatment had three biological replicates with 500 seeds per replicate. All the biological replicates were from the same seed stock. The sample was frozen in liquid nitrogen and stored in a − 80 °C refrigerator for transcriptome sequencing and the determination of physiological indices.

### Determination of seed TTF, ZT, GA_3_, and ABA contents in seeds

Seed vigor was determined by the 2,3,5-triphenyltetrazolium chloride (TTC) method, which measured TTF contents. Oxidized colorless TTC produces hydrogen from dehydrogenase in the living cell tissues of seed embryos, and TTC in seed embryos is reduced to red TTF by hydrogen [91]. Other physiological indicators were determined by grinding seeds after liquid nitrogen treatment. Soluble sugar content was measured by anthrone colorimetry [92]. The contents of ZT, GA_3_, and ABA contents were measured as described by Ding et al. [93].

### Library construction and transcriptome sequencing

The total RNA of *F. hupehensis* seeds was extracted using a TAKARA MIniBEST Plant RNA Extraction Kit in accordance with the manufacturer’s instructions. RNA samples were tested for degradation and impurities by using 1% agarose electrophoresis. Sample purity was measured using a NanoDrop 2000 microspectrophotometer (IMPLEN, CA, USA), and the integrity and concentration of the RNA sample were detected using an Agilent 2100 RNA Nano 6000 Assay Kit (Agilent Technologies, CA, USA). Sequencing libraries were obtained using the NEBNext1Ultra™ RNA Library Prep Kit for Illumina* (NEB, USA) in accordance with the manufacturer’s instructions. In brief, total mRNA was isolated with Oligo (dT), broken into short fragments, and then used to synthesize the first strand of cDNA. The purified double-stranded cDNA was subjected to terminal repair, addition of base A, and sequencing ligation. Finally, target-size fragments were isolated for PCR amplification to complete the construction of nine cDNA libraries. The concentration and quality of these libraries were tested using Agilent 2100 and Qubit 2.0, respectively. The nine cDNA libraries were then sequenced by Annoroad Gene Technology Corporation (Beijing, China) using Illumina HiSeq 2500.

### De novo assembly and functional annotation of unigenes

The original sequence data were obtained by Illumina HiSeq sequencing. High-quality reads (clean reads) were obtained by removing low-quality reads (bases with a mass value of Q ≤ 19, accounting for over 15% of the total bases), joint-pollution reads (the number of bases contaminated by the linker in reads is greater than 5 bp), and reads with N ratios greater than 5%. All clean reads of the nine libraries were obtained using Trinity software and de Bruijn method to assemble the full-length transcript sequences [94], and the longest transcripts of each gene were considered as unigenes based on the transcripts. The transcriptome assembly sequence was annotated with Trinotate, and functional annotations were performed using the databases PFAM (protein domain identification), Nr (NCBI nonredundant protein sequences), Swiss-Prot (a manually annotated and reviewed protein sequence database), GO annotation, COG annotation, and KEGG. PlantTFDB (Plant Transcription Factor Database) was used to annotate TFs.

### DEG analysis

RPKM value were used to represent the expression abundance of reads corresponding to Unigenes. In this study, we used DEseq242 to compare the treatment group with the reference group and selected | log 2 Ratio | > 1 and Q < 0.05 genes as DEGs [95]. Enrichment analysis of DEGs was analyzed using the GO and KEGG databases to obtain a detailed description of the DEGs during seed germination. The DEGs were clustered with *p* < 0.05, which indicated that the cluster distribution was significant.

### qRT-PCR analysis

DEGs were selected for qRT-PCR analysis, and *18S* was used as the internal reference gene. Specific primers were designed based on the sequence of unigenes using Primer 5.0 and are listed in Table S4. The RNA extracted from each sample (600 ng) was used to synthesize single-strand cDNA with a PrimeScript RT Reagent Kit following the manufacturer’s instructions. A LineGene 9600 Plus real-time PCR instrument (Bori, Hangzhou) was used to perform PCR. The relative quantitative expression of the genes was calculated using the 2^-ΔΔCt^ method [96]. Each sample was prepared in triplicate (500 seeds per replicate). The normalized values of relative expression and RPKM values were calculated using log2 fold variation measurements, and the correlation between RNA-seq and qPCR results was analyzed using these values.

### Statistical analysis of data

Data were analyzed using Excel and SPSS by ANOVA followed by Tukey’s significant difference test at *p* ≤ 0.05. All data had three biological repeats.

## Additional files

Additional file 1: **Table S1.** Evaluation of sample sequencing data. (XLSX 9 kb)

Additional file 2: **Table S2.** All DEGs from the transcriptome of *F. hupehensis* seeds. (XLSX 5488 kb)

Additional file 3: **Figure S1.** Correlation coefficients among the RPKM of unigenes of the samples. All data shown indicate the results of three biological replicates (*n* = 3). (PDF 1132 kb)

Additional file 4: **Figure S2.** Clusters of DEGs obtained by K-means. DEGs were divided into eight subclasses. All data shown reflect the results of three biological replicates (*n* = 3). (PDF 977 kb)

Additional file 5: **Figure S3.** Hierarchical clustering of DEGs between the W and CK groups, the G and CK groups, and the G and W groups. All data shown indicate the results of three biological replicates (*n* = 3). The sample names are shown at the bottom of the figure. Changes in expression level are indicated by a change in color; blue indicates a lower expression level, whereas red indicates a higher expression level. (PDF 5411 kb)

Additional file 6: **Table S3.** Key DEGs related to *F. hupehensis* seed germination. (XLSX 17 kb)

Additional file 7: **Table S4.** Differentially expressed TFs related to *F. hupehensis* seed germination. (XLSX 11 kb)

Additional file 8: **Table S5.** Primers used in qRT-PCR. (XLSX 12 kb)

## Abbreviations

AAO3: Abscisic-aldehyde oxidase
ABA: Abscisic acid
ABA2: Xanthoxin dehydrogenase
ABF: ABA responsive element binding factor
ABI5.5: ABSCISIC ACID-INSENSITIVE 5 like 5
ABI5.6: ABSCISIC ACID-INSENSITIVE 5 like 6
ALDO: Fructose-bisphosphate aldolase, class I
B-ARR: Two-component response regulator ARR-B family
BPGM: Bisphosphoglycerate/phosphoglycerate mutase
CAT: Catalase
CCR4-NOT: CCR4-nontranscriptional complex
CKX: Cytokinin dehydrogenase
CPS: Copalyl pyrophosphate synthase
CRE1: Arabidopsis histidine kinase 2/3/4 (cytokinin receptor)
CYP450: Cytochrome P450 monooxygenase 1
CYP707A: (+)-Abscisic acid 8′-hydroxylase
CYP735A: Cytokinin trans-hydroxylase
DEGs: Differentially expressed genes
Edcp: Enhancer of mRNA-decapping protein
ENO: Enolase
FBP: Fructose-1,6-bisphosphatase I
GA_20OX_: GA_20_ oxidase
GA_3_: Gibberellin
GA_3_ox: GA_3_ oxidase
GAI1: DELLA protein GAI1
GAP2: Glyceraldehyde-3-phosphate dehydrogenase (NAD(P)
GAPDH: Glyceraldehyde 3-phosphate dehydrogenase
GAPN: Glyceraldehyde-3-phosphate dehydrogenase (NADP+)
GAPOR: Glyceraldehyde-3-phosphate dehydrogenase (ferredoxin)
GCL: Glutamate-cysteine ligase
GID1: Gibberellin receptor
GPI: Glucose-6-phosphate isomerase
GST: Glutathione S-transferase
IPT: Adenylate isopentenyltransferase
KAO: Ent-kaurenoic acid monooxygenase
LSm4: U6 snRNA-associated Sm-like protein LSm4
NCED: 9-cis-epoxycarotenoid dioxygenase
PDHB: Pyruvate dehydrogenase E1 component beta subunit
PFK: 6-phosphofructo-2-kinase
PfkC: ADP-dependent phosphofructokinase/glucokinase
PFP: Diphosphate-dependent phosphofructokinase
PGAM: 2,3-bisphosphoglycerate-dependent phosphoglycerate mutase
PGK: Phosphoglycerate kinase
PGM: Phosphoglucomutase
PK: Pyruvate kinase
POD: Peroxiredoxin
PP2C: Protein phosphatase 2C
PVP: Polyvinylpyrrolidone
PYR/PYL: Abscisic acid receptor PYR/PYL family
QPMI: 2,3-bisphosphoglycerate-independent phosphoglycerate mutase
SLY/DID2: F-box protein GID2
SnRK2: Serine/threonine-protein kinase SRK2
SOD: Superoxide dismutase
TCA cycle: Tricarboxylic acid cycle
TF: Phytochrome-interacting factor 4
TFs: Transcription factors
TTC: 2,3,5-Triphenyltetrazolium chloride
TTF: Triphenylformazan
XRN2: 5′-3′ exoribonuclease
ZEP: Zeaxanthin epoxidase
ZOG: Zeatin O-glucosyltransferase
ZT: Zeatin
