# Supplementary figures and images for: Comparative transcriptome analysis revealing the potential mechanism of seed germination stimulated by exogenous gibberellin in Fraxinus hupehensis
Source: BMC Plant Biol. 2019 May 15;19:199. doi: 10.1186/s12870-019-1801-3 (PMC6521437; doi:10.1186/s12870-019-1801-3)

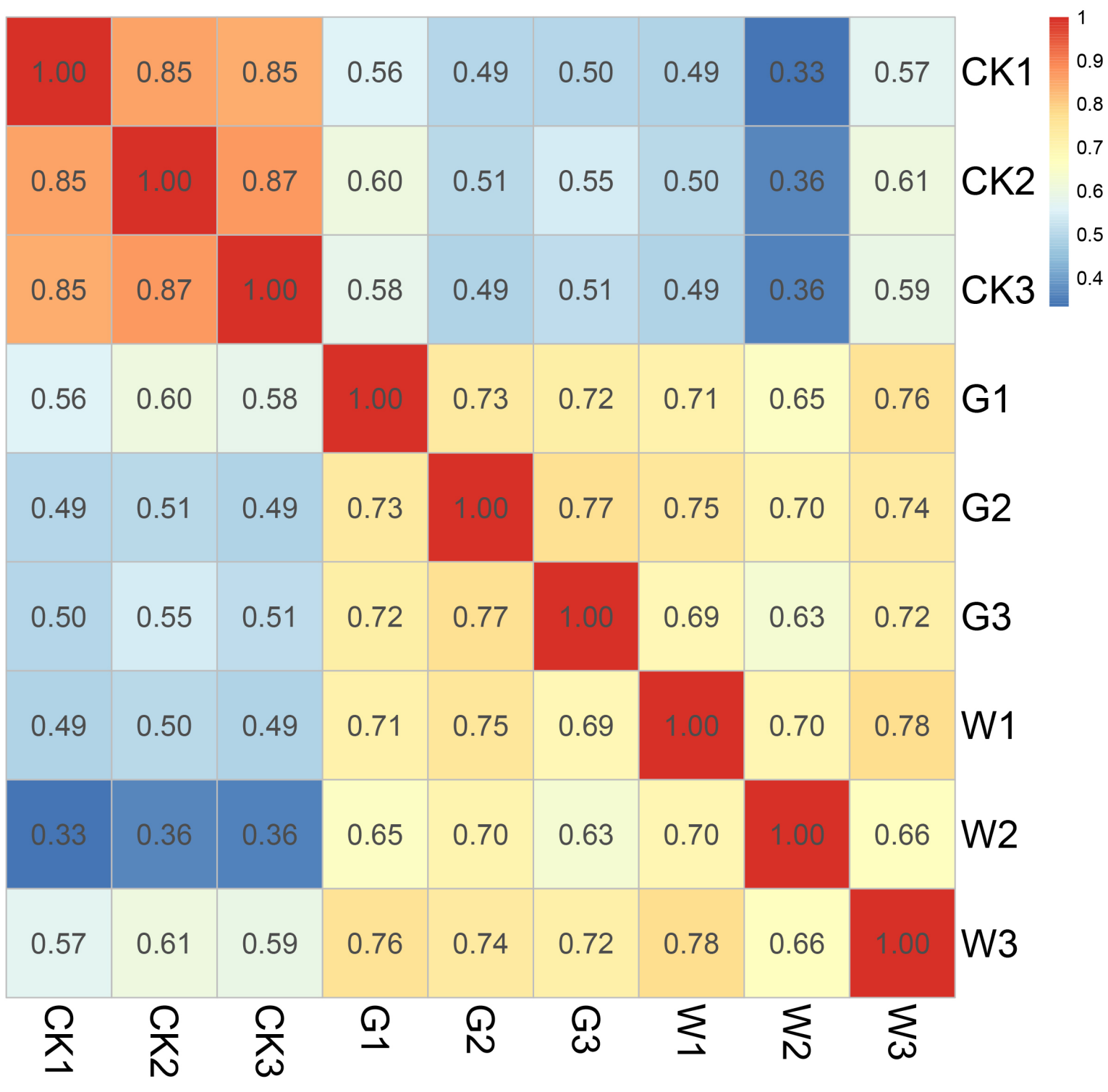

Supplement: Supplementary file 3 — Figure S1. Correlation coefficients among the RPKM of unigenes of the samples. All data shown indicate the results of three biological replicates (n = 3). (PDF 1132 kb) [file 12870_2019_1801_MOESM3_ESM.pdf]

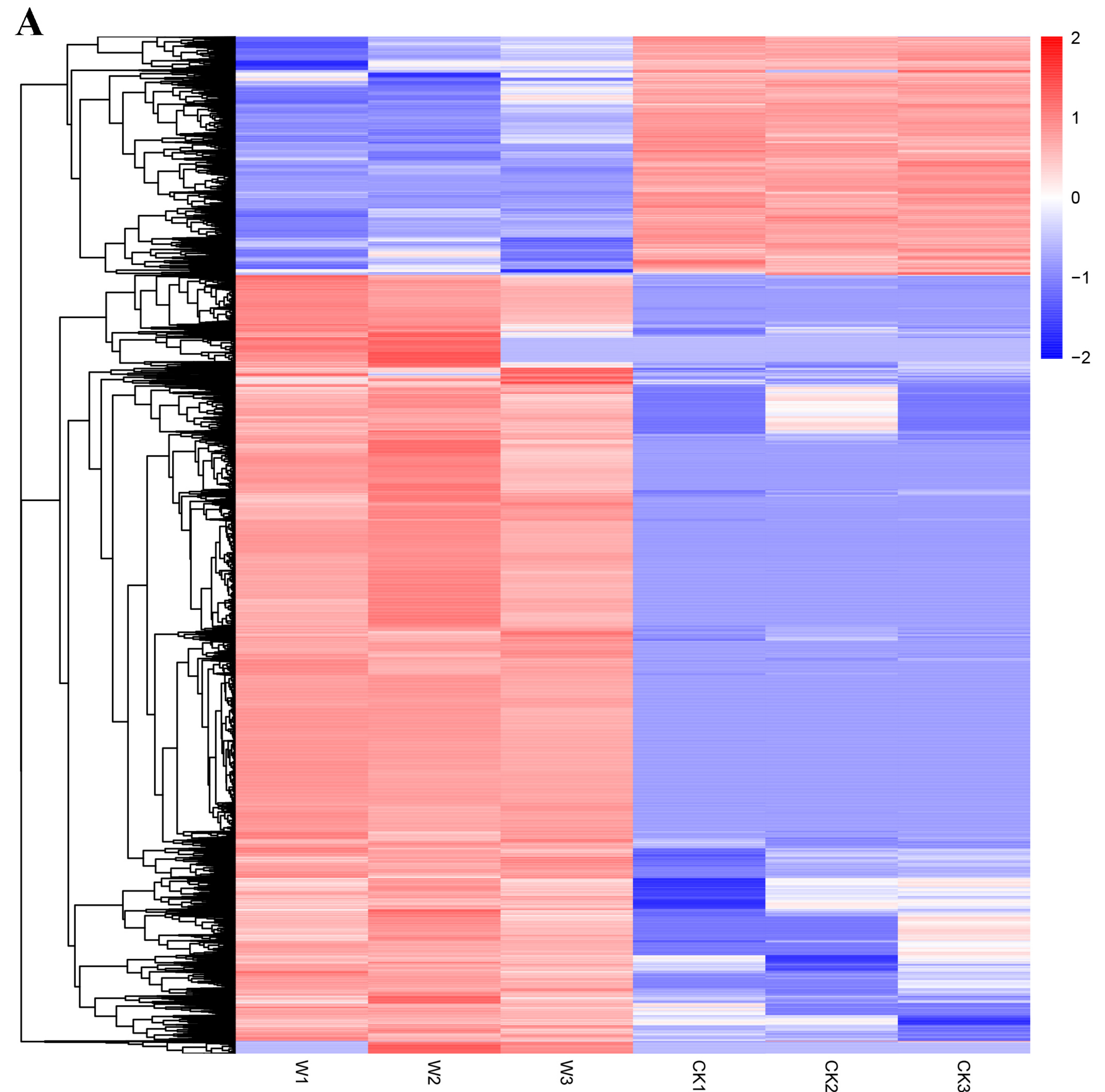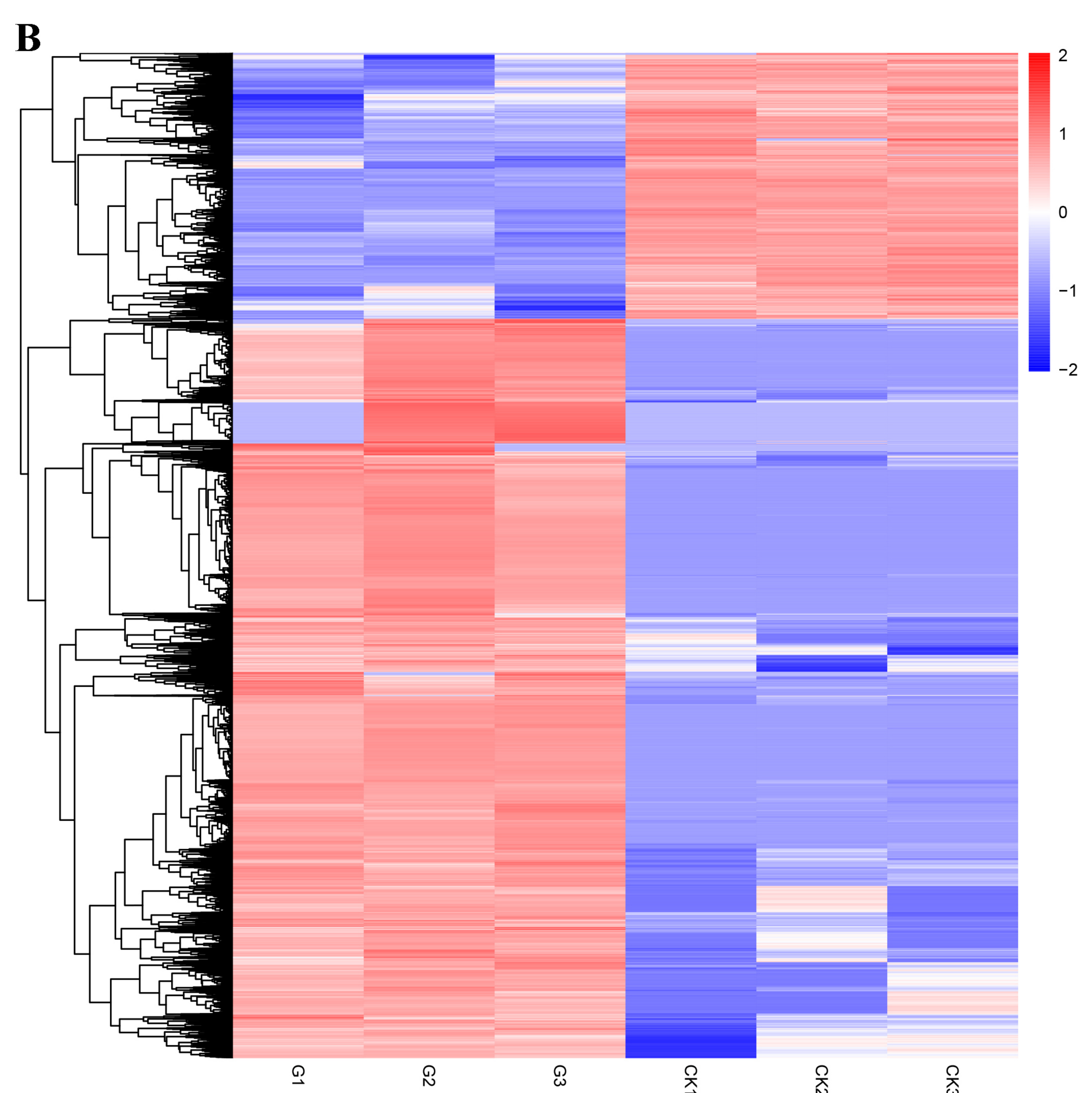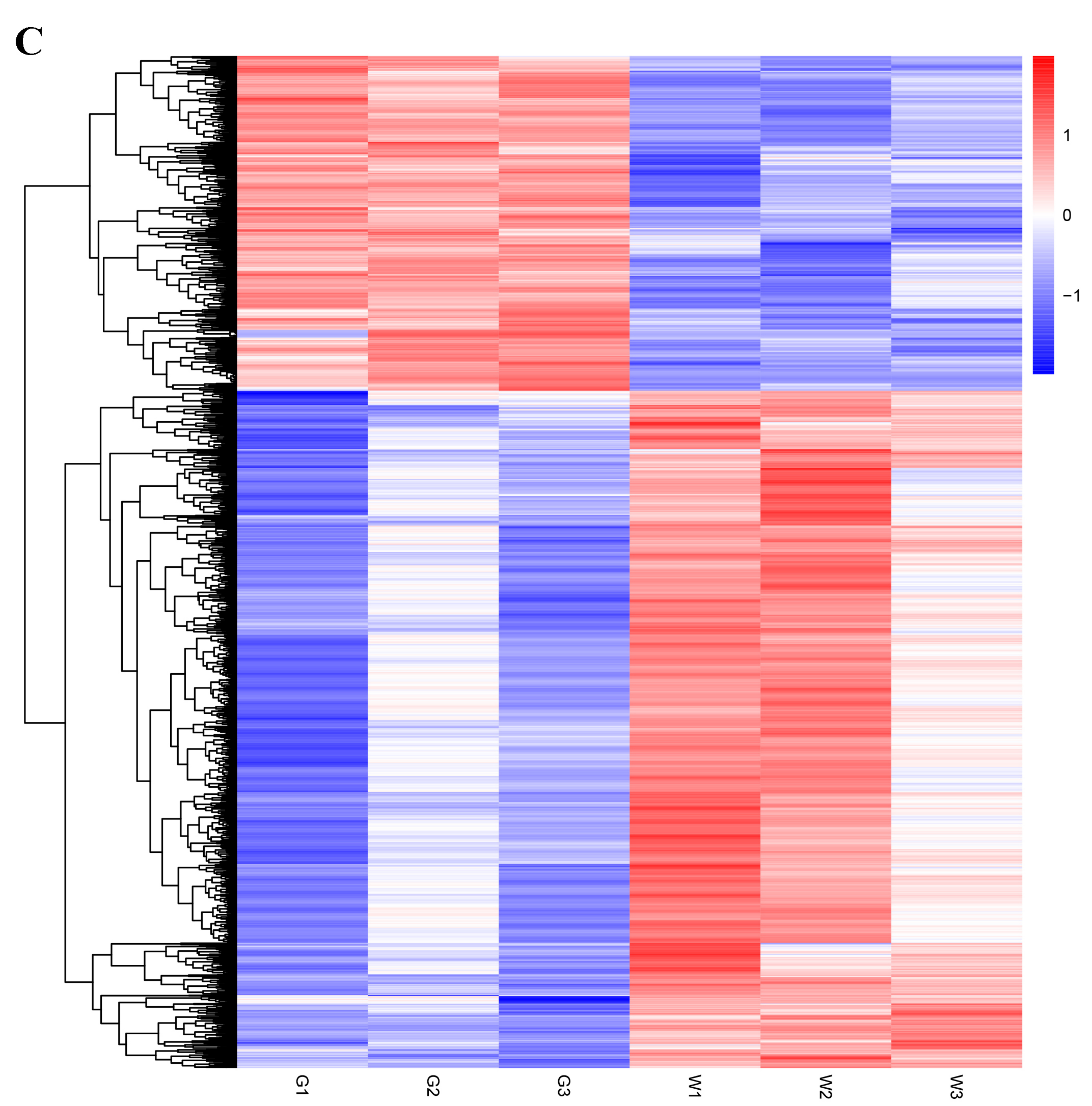

Supplement: Supplementary file 5 — Figure S3. Hierarchical clustering of DEGs between the W and CK groups, the G and CK groups, and the G and W groups. All data shown indicate the results of three biological replicates (n = 3). The sample names are shown at the bottom of the figure. Changes in expression level are indicated by a change in color; blue indicates a lower expression level, whereas red indicates a higher expression level. (PDF 5411 kb) [file 12870_2019_1801_MOESM5_ESM.pdf]
