# Supplementary material for: Comparative transcriptome analysis revealing the potential mechanism of seed germination stimulated by exogenous gibberellin in Fraxinus hupehensis
Source: BMC Plant Biol. 2019 May 15;19:199. doi: 10.1186/s12870-019-1801-3 (PMC6521437; doi:10.1186/s12870-019-1801-3)
Supplement: Supplementary file 4 — Figure S2. Clusters of DEGs obtained by K-means. DEGs were divided into eight subclasses. All data shown reflect the results of three biological replicates (n = 3). (PDF 977 kb) [file 12870_2019_1801_MOESM4_ESM.pdf]

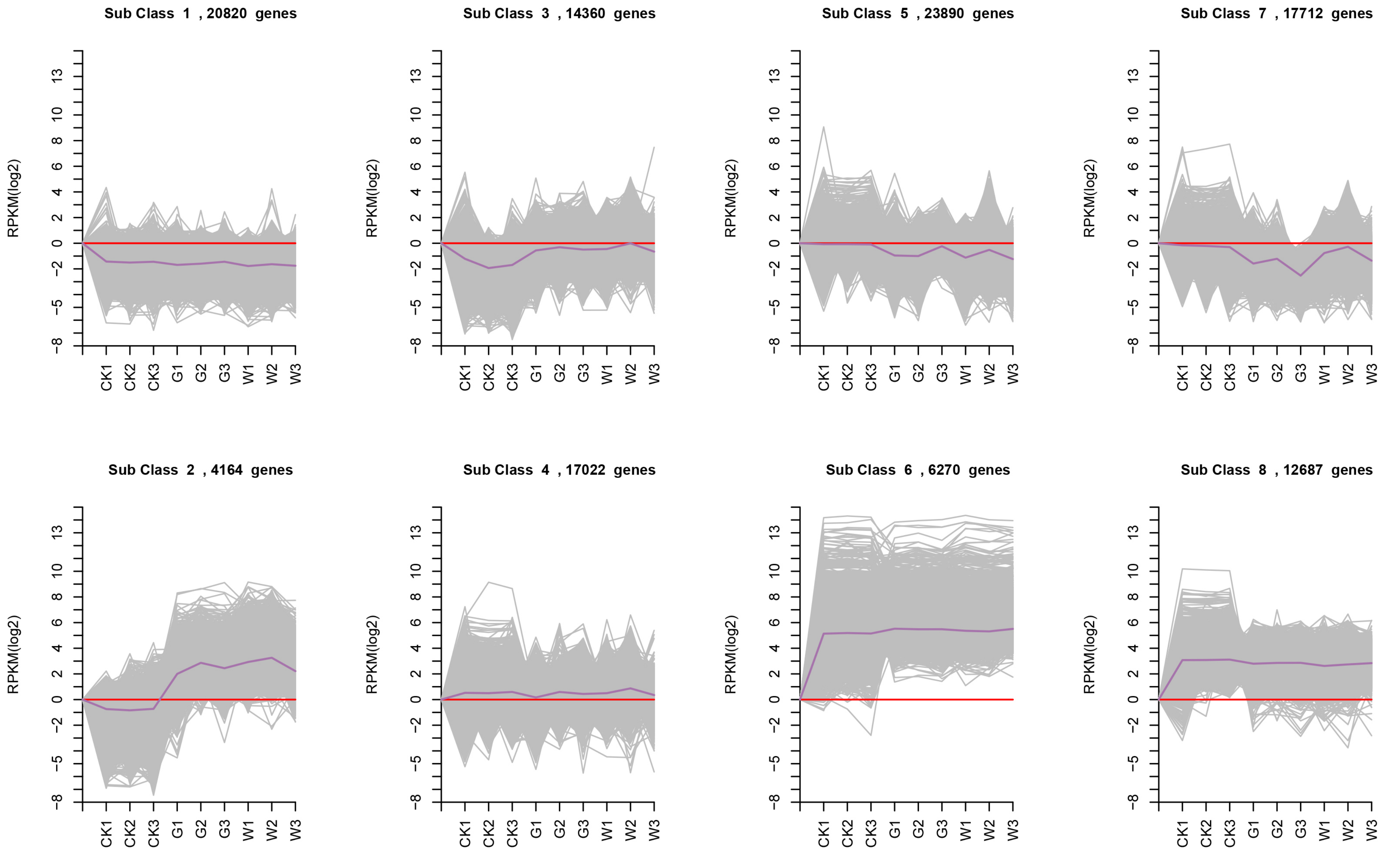
Sub Class 1 , 20820 genes
Sub Class 3 , 14360 genes
Sub Class 5 , 23890 genes
Sub Class 7 , 17712 genes
Sub Class 2 , 4164 genes
Sub Class 4 , 17022 genes
Sub Class 6 , 6270 genes
Sub Class 8 , 12687 genes
RPKM(log2)
CK1 CK2 CK3 G1 G2 G3 W1 W2 W3
